# Why Real-World Problems Go Unresolved and What We Can Do about It: Inferences from a Limited-Resource Model of Successful Intelligence †

**DOI:** 10.3390/jintelligence6030044

**Published:** 2018-09-13

**Authors:** Robert J. Sternberg

**Affiliations:** Department of Human Development, College of Human Ecology, Ithaca, NY 14853, USA; robert.sternberg@cornell.edu

**Keywords:** components, creativity, inoculation, intelligence, limited-resource model, wisdom

## Abstract

In this article I suggest why a symposium is desirable on the topic of why, despite worldwide increases in IQ since the beginning of the 20th century, there are so many unresolved and dramatic problems in the world. I briefly discuss what some of these problems are, and the paradox of people with higher IQs not only being unable to solve them, but in some cases people being unwilling to address them. I suggest that higher IQ is not always highly relevant to the problems, and in some cases, may displace other skills that better would apply to the solution of the problems. I present a limited-resource model as an adjunct to the augmented theory of successful intelligence. The model suggests that increasing societal emphases on analytical abilities have displaced development and utilization of other skills, especially creative, practical, and wisdom-based ones, that better could be applied to serious world problems. I also discuss the importance of cognitive inoculation against unscrupulous and sometimes malevolent attempts to change belief systems.

## 1. Introduction

The essays in this special section of the *Journal of Intelligence* deal with why intelligence, defined narrowly, is not sufficient for creating a better world and may even lead to a worse world. They also address the more important question: What can we do about it? In this editorial, I present here some of my personal views on why IQ is not sufficient for creating a better world—these views, I believe, are consistent with but also extend beyond conclusions drawn directly from empirical research. Here is the conundrum: IQs rose 30+ points in the 20th century, but serious world problems remain and in some cases are getting worse. For example, air pollution around the world is so bad that it is cutting a year, on average, off people’s lives [1]. Income inequality is bad and growing worse [2], leading to populist movements [3] and an increased likelihood of irresponsible governments that cater to whims rather than to reason. Attempts at global denuclearization of weapons largely have failed [4], leaving the world susceptible to the vagaries of deteriorating international relations, especially with regard to North Korea and Iran.

If people are so smart, why can they not solve or often even begin realistically to address the most pressing problems the world is facing? I propose in this article a model to explain why increasing IQs actually may decrease the probability of solving any of these problems. Although the Flynn effect may now be reversing in some locales [5], substantial increases in IQ from the very beginning of the twentieth century remain.

## 2. A Limited-Resource Model

According to the theory of successful intelligence, intelligence at its base comprises a series of information-processing components acting upon mental representations in a variety of environmental contexts [6]. These components are of three kinds: metacomponents, performance components, and knowledge-acquisition components. Metacomponents are used to plan, monitor, and evaluate reasoning, problem solving, and decision making. Performance components are used to execute these higher order cognitive tasks. Knowledge-acquisition components are used to learn how to do these tasks in the first place (see Note 6).

The three kinds of components, according to the original triarchic theory of intelligence and its later manifestation as the theory of successful intelligence, produce three basic kinds of mental functioning and a subsidiary one. Creative intelligence is involved when the components are applied to tasks and situations that are relatively (but not completely) novel. Analytical intelligence is involved when the components are applied to fairly abstract but nevertheless relatively familiar kinds of problems, such as the ones students encounter in school. And practical intelligence is involved when the components are applied to relatively concrete and familiar kinds of problems, such as the challenges of everyday life [7]. The key point here is that the same information-processing components are involved in creative, analytical, and practical thinking—what differs is how they are applied. A fourth kind of thinking, wise thinking, is involved when the components engage in creative, analytical, and practical thinking to help achieve a common good, over the long as well as the short term [8,9].

The theory as originally proposed did not consider resource constraints. That is, what cognitive limitations are there in the generation of components to accomplish the tasks necessary for one’s life? Research would suggest that there are three major sets of constraints, or at least, cognitive ones. The first set comprises working-memory constraints [10,11]. One can do only as many tasks as one can hold in one’s working memory. A second set of constraints is attentional [12,13]. To execute a task, one needs to attend to it. And to execute it well, one needs to ensure that one’s attention is directed properly to the various elements of the task. A third set of constraints is speed-related [14,15]. The faster one mentally processes information, the more components one can execute in a given amount of time. Of course, speed says nothing about the accuracy with which the components will be executed, and accuracy matters at least as much as speed [16].

These constraints (and doubtless, others) are important because they limit an individual’s ability to execute components in intelligent reasoning, decision making, and problem solving. That is, one’s ability to solve problems and accomplish life tasks will depend on one’s componential skills, on the one hand, and one’s cognitive constraints (working memory, attention, mental speed) for applying them.

## 3. Effects of Societal Context

At different points in the development of a society and the sociocultural context in which it is embedded, rewards for different kinds of thinking and behaving will differ. For example, Renaissance Florence saw an explosion of creative intelligence because of the patron system and because of mutual reinforcement of artists for each other’s work. After the Soviet Union’s launch of Sputnik, creative work in space engineering exploded because of societal needs. What kind(s) of abilities are most being rewarded today? Let’s look at where the rewards are?

I argued as early as 1985 that US society, and perhaps other societies as well, were tipping the reward system very heavily toward analytical uses of components (see Note 6). That trend has accelerated greatly as a result of several societal trends.

### 3.1. Rising IQs

As IQs rose in the 20th century, the memory and analytical skills contributing to them increased and society saw some gains from these enhanced skills. People became better able to understand and use technology, for example—cell phones, computers, even everyday appliances connected to the Internet. Just as tall people are more likely to play basketball, or attractive people more likely to model, high-IQ people are more likely to find jobs that match their considerable intellectual skills. As IQs rose, people needed them more, just as when people have grown taller as a result of better nutrition, society took these heights into account in designing furniture, vehicles of conveyance, and the like. Those who did not participate in the growth may have found more and more resources simply physically out of reach. Short people today have trouble reaching many things, but those people may be average or even above average relative to heights just a century ago, in the same way that many of the lower IQ people of today would have been above average a century ago.

### 3.2. Standardized Testing

Standardized testing as done in the United States and in many other countries very heavily emphasizes memory and analytical skills, largely to the exclusion of creative, practical, and wisdom-based skills [17]. The items used assess accumulated knowledge and the ability to analyze that knowledge, but stop there [18]. Those are useful skills. But the tests do not measure students’ abilities to come up with new ideas, to apply those new ideas in practical settings, or to ensure that their ideas help to achieve some kind of common good [19,20]. So students are admitted to private schools, gifted programs, and institutions of higher education largely on the basis of their memory and analytical skills, skills that are useful but inadequate in and of themselves for solving serious world problems. 

Once a certain group starts to dominate in an institutional setting, self-fulfilling prophecies tend to take root. People expect more from the people identified as “winners,” and they get more. The result is that those people expected to be successful become successful, and the preference for like people passes on from one generation to the next. The next generation, in evaluating people, looks for others more or less like themselves, leading to whatever traits were valuable before to matter even more (see Note 6).

### 3.3. Instruction in Schools

Because school curricula, at least today, are so heavily geared toward whatever it is that standardized tests measure, instruction in schools has become in its emphases, as it has been for a long time, much like the tests. Instruction emphasizes the same memory and analytical abilities that the tests test. The tests and the schools reinforce each other in an unending loop. Indeed, Alfred Binet created the IQ test to reflect skills needed for success in school, and schools teach to the kinds of skills that his test and others like it measure. This is not in itself a bad thing, of course, but it may crowd out other kinds of skills that also matter, such as creative, practical, and wisdom-based ones.

### 3.4. Social Media

Social media undoubtedly have served some useful functions in connecting people who otherwise would never have been connected. And they have provided a way for people to reach out with their ideas in the past who never could have had any audience. But social media bypass a crucial part of the creative process—refereeing. The result is that people often pretty much say whatever occurs to them. This is bad for analytical-skill development and utilization and even worse for creative-skill development and utilization. Creativity requires critique of whether a novel idea is good and may serve some positive function. Because negative and even untrue postings spread faster on social media than do positive and true postings [21], the reinforcement system encourages only a low level of the serious analytical scrutiny that is necessary for the entire creative process. And because wisdom requires reflection and careful scrutiny of what one says, social media’s emphasis on quick and unreflective responses almost certainly discourage wise thinking.

Even worse, the reinforcement system of social media is oriented toward making money for advertisers, as that is the source of income for the companies that produce social media. Advertising today, as always, works best when it bypasses critical thinking. There is nothing new in all this. Even in the 1950s, skeptics were concerned about how the medium of the moment–television–bypassed critical thinking and allowed advertisers to get a toehold on a person’s consciousness [22]. Social media and many Internet applications are designed to free users from cognitive inhibitions—that is what advertising is about–and in the process, they have degraded the thinking even of people with high levels of intelligence [23,24]. 

People who are intelligent may believe that they are immune to any potential dumbing-down effects of social media. But a number of researchers have argued the opposite—that people’s belief that they are immune to foolish or mindless work actually makes them become more susceptible to it [25,26,27,28,29]. They may be intelligent as a “trait,” but even people who are more intelligent as a trait may behave foolishly as a state—that is, they can fall for the same tricks as others, maybe more so because they believe they cannot.

### 3.5. Surveillance

We live, more and more, in surveillance societies [29,30,31]. In parts of the world, surveillance is so tight that one can be viewed as having little more privacy than in George Orwell’s “*1984”* [32]. In some places, it is hard to walk anywhere in a public space without cameras observing one’s movements. One’s use of the Internet is closely monitored by companies such as Facebook and Google, as well as by advertisers looking to get an edge in sales. 

The surveillance is not all from the top-down. People are doing more surveillance on each other. Some of it is probably good, as in the open-science movement, designed to achieve greater transparency in scientific research. But the cost of such a movement is more surveillance of scientists by one another, with increased emphasis, again, on analytical processing at the expense of creative and perhaps wisdom-related information processing. The amount of registration and paperwork increases, leaving potentially less time for creative work [33]. And current emphasis on replication has the same effect, leading researchers to spend time processing information analytically—to be sure of exactly replicating someone else’s work—rather than going much beyond what has been done before. Indeed, in a strict replication, the idea is *not* to go beyond what was done before—to replicate methods as exactly as possible [34]. A conceptual replication goes a bit beyond the past, but is a limiting form of creative enterprise [35]. On the positive side, of course, science becomes more open and transparent. But the emphasis on analytical processing, once again, increases, in a limited-resource model, at the possible expense of creative processing in generating novel and useful ideas.

Of course, many other forces operate in society, but the point, I believe, is clear. Many societies in the world, including that in the United States, have come more and more to reward memory and analytical skills. I believe there are exceptions, such as the culture of Silicon Valley, which also emphasizes creative and, to an extent, practical skills. But the isolation of Silicon Valley from much of the rest of the United States bespeaks the cultural difference between it and much of the rest of the country.

If, indeed, resources are limited, then the emphasis placed on memory and analytical skills will tend to crowd out the development of creative and practical skills. The result will be successive generations of students who become increasingly analytical in their focus, in the mode of thinking required for problems on IQ tests and related tests, such as the SAT and ACT. It should be noted that this analytical focus is quite different from the focus of rational thinking. That is, one can have a high IQ and be lacking in rational and critical thinking capacities of the kinds needed for success in everyday life [36,37,38]. So smart people not only can be foolish (lacking in wisdom for the common good), but also, lacking in rationality.

## 4. Interaction of Components of Intelligence with Societal Context

To return to the original question that motivated this essay: If intelligence is truly important to real-world adaptation, and IQs have risen 30+ points in the past century, then why are there so many unresolved and dramatic problems in the world today, and what can be done about it?

I have argued that a number of societal forces have conspired with the limited resources of the human brain to emphasize some skills over others, namely, memory and analytical skills at the potential expense of creative, practical (common-sense), and wisdom-based skills. Individuals start with a wide range of potentials for the development of cognitive skills. Their own predispositions plus societal forces then result in the greater development of some and the lesser development of others into competencies. And then, again, their predispositions and societal forces lead some people to become experts of one kind or another [39,40,41]. In many of our societies, students simply are not developing the creative skills they need for success in solving difficult world problems [42], nor the practical nor the wisdom-based skills either. Like the carpenter who has a hammer and looks for things to be hammered, they look for problems they can solve by memory or analytically, which often means that they will be looking for things, ideas, and people to critique more than they will be looking for things and ideas to create in common-sense ways so that they help to achieve a common good. In essence, we risk becoming a society of replicators and critics (see Note 6).

Fortunately, there are many forces that also encourage creativity—competiveness in most fields (from research to industry), rapid changes in social customs, rapid changes in the technology we can creatively exploit to get our work done, and sometimes even rare work environments that actually encourage creative thinking rather than merely saying they do. But the greatest problem is that that there are few forces that encourage the development of wisdom—using our creative, analytical, and practical skills to seek a common good. 

For most of its history, the United States was a country that sought a “common good.” Unfortunately, the common good was not quite “common”—it was almost exclusively for white people, mostly for males, and often excluded people of non-Christian religions, and even at times excluded Catholics. The Civil Rights movement of the 1960s and 1970s helped achieve something more nearly approaching equality of various groups, but what recent times have made clear is that it did so at a cost: Many people who before had been in the privileged class—even if they were not economically well off—felt that their rightful place was either being stolen from them, or else, that it was being too widely shared. Partly as a result, the United States is highly polarized today, perhaps as never before since the Civil War. Similar problems have arisen in in Europe and beyond. Those who once felt themselves to be “have’s” came to feel like “have-not’s,” and through populist movements are seeking to become have’s again (see Note 3).

The only viable solution to this problem is for people to use their creative, analytic, and practical intelligence wisely—toward a common good. But the forces described above—testing, schooling, social media, surveillance—all lead people away from this goal. And the individualism of many of our societies, combined with cultural clashes within and between those societies, have made it difficult for people to see beyond their own tribal interests [43]. If there is one thing schools are not doing, however, it is teaching for wisdom [44], arguably the most important element in the augmented theory of successful intelligence [45]. Teachers do not know how and because wisdom is not tested, it remains nearly invisible in the schooling process. 

On top of this, the intelligence field, after perhaps a brief flirtation with broader theories of intelligence such as those of Gardner [46] and Sternberg [45], seems to be moving back toward its traditional position in favor of narrower psychometrically-based views [47,48,49] that, while multifaceted, still view intelligence as having general ability, or *g* at the top of a hierarchy of abilities. Some argue for the psychometric model above other models [50] and others argue that the task of intelligence research is simply to understand the biology of *g* and related constructs [51].

All of this might not matter if the state of the world were not of such great concern at the moment. Perhaps people always feel that things are precarious. But the return of somewhat mindless populism [52], as existed before World War II, has to be a major concern to a world in which democracy is on the decline [53], where income inequality favoring those with high IQ is feeding resentment the very populism that is threatening democracy [54], and where air pollution is not only shortening life spans, but apparently lowering global intelligence [55].

## 5. Failure of Cognitive Inoculation

Professor William McGuire was a social psychologist and, in particular, a cognitive-consistency theorist. But in retrospect, he might be viewed as well as a distinguished theorist of practical intelligence. He proposed an inoculation theory, according to which people could be helped to maintain sensible and cognitively consistent beliefs if they were “inoculated” against (sometimes malevolent) attempts to change these beliefs [56,57,58]. The idea was much like physical inoculation: By challenging people’s beliefs with weak counterarguments, one could inoculate their cognitive systems against later stronger (and sometimes less than rational) counterarguments.

Of course, sometimes people do need to change their beliefs. McGuire’s concern was, however, as is that here, with attempts to change beliefs that people need to maintain clear and cognitively consistent thinking. Demagogues in all domains (not just political—business, religion, science, whatever) attempt to change beliefs by repeating, over and over, powerful and often simplistic belief-changing statements. They recruit allies to assist them in their attempts to overthrow reason. No one is immune to such forces, least of all the “smart” people who may believe they are immune and thus may put up little resistance. 

According to McGuire, four elements are necessary for inoculation to occur:

1. *Threat.* The individual must recognize that there is a threat to his or her considered beliefs. In our case, the threat might be to the view that democracy is desirable and that people should have equal rights.
2. *Refutational preemption.* The individual must be able to activate his or her own arguments and strengthen those beliefs through countering threats to the beliefs.
3. *Delay.* As with a biological inoculation, people need some time, in this case after the presentation of the weak counterarguments, for cognitive inoculation to work. Perhaps people use that time to reflect upon their views and why they are solid.
4. *Involvement.* People need to have sufficient involvement in the issue at hand to want to defend their beliefs and to activate them in the face of threat to them. Otherwise, they may not care enough to counter attacks on their beliefs.

Again, some beliefs of course need changing. None of us should view our beliefs as sacrosanct. Nothing said here stands against that basic fact.

Yet, today, fundamental beliefs are under attack. It often is hard to figure out what one or another political party in a given country stands for. How could beliefs change so quickly? There is good reason to believe that cognitive inoculation today is at threat. The main reason is perhaps that people are just not ready to apply cognitive inoculation to new media, such as the Internet and in particular the social media through which so much new information is now presented. People just have not had a chance to adjust. People of course need some time to adjust to any new medium, even newspapers or television. But going back just a decade or two, most media, at least in democracies, made at least some attempt to present balanced news. In the United States, whether one listened on the television of the 1960s to Chet Huntley and David Brinkley (NBC), Walter Cronkite (CBS), or Howard K. Smith (ABC), one received basically the same news. Today that is no longer true. The world as presented by Fox is unrecognizable to viewers of (or listeners to) CNN, and vice versa. The Internet allows people and clever social-media providers to screen out what particular people don’t want to hear, with the result that they live in a mini-universe of like-minded people. They find it hard to understand why anyone would disagree [59]. 

Biologically, people actually have two immune systems, not just one [60]. The older immune system is a general-purpose one, which can protect us against generalized biological (bacterial, viral, parasitic, etc.) threats. The newer immune system specifically targets particular new threats that the body has not previously mobilized specific mechanisms to defend against. The problem is that the new immune system is slower to act, because it needs to recruit new antibodies to guard against and attack the specific threats. In the meantime, while the need for antibodies is being assessed, the antibodies are being created, and then the antibodies are being mobilized, the challenge to the body can defeat it, or render it permanently incapacitated. 

I suggest that the cognitive immune system may have the same challenge as the biological one. Some threats to our cognitive system, such as when someone tries to cheat us in an interpersonal interaction, may stimulate our “old” cognitive immune system. That is, we are used to such threats and know how to handle them. People have been dealing with such challenges over the millennia. But the newer threats—such as via social media—invoke much less of a hard-wired response, and that response is probably generalized and weak, as would be our biological response to a new threat. By the time the newer cognitive immune system is recruited, it may or may not be too late. Our minds may already be “infected” with maladaptive “viruses” by the time the new cognitive immune system sets to work, and by then, we may not be able to marshal sufficient cognitive resistance to change what have become maladaptive modes of thought. Thus can we be beguiled by would-be despots and their henchmen.

It is useful that intelligence theorists are studying intelligence in the laboratory using biological, reaction-time, and other related methods. But our ability to direct our intelligence to face the challenges of modern society is under threat. Is it not time for intelligence theorists to take seriously threats that are themselves serious to the well-being of civilization? The essays in this special section of the *Journal of Intelligence* consider current challenges to, and problems in the world, and how intelligence, broadly considered, can help us better to face and perhaps even solve them.
